# Differential Effect of Generalized and Abdominal Obesity on the Development and Progression of Diabetic Retinopathy in Chinese Adults With Type 2 Diabetes

**DOI:** 10.3389/fmed.2022.774216

**Published:** 2022-05-27

**Authors:** Xiaoyan Han, Huimin Wu, Youjia Li, Meng Yuan, Xia Gong, Xiao Guo, Rongqiang Tan, Ming Xie, Xiaoling Liang, Wenyong Huang, Hua Liu, Lanhua Wang

**Affiliations:** ^1^The First People's Hospital of Zhaoqing, Zhaoqing, China; ^2^Shenzhen Children's Hospital, Shenzhen, China; ^3^State Key Laboratory of Ophthalmology, Zhongshan Ophthalmic Center, Sun Yat-sen University, Guangdong Provincial Key Laboratory of Ophthalmology and Visual Science, Guangdong Provincial Clinical Research Center for Ocular Disease, Guangzhou, China; ^4^Department of Ophthalmology, Third Affiliated Hospital of Jinzhou Medical University, Jinzhou, China

**Keywords:** generalized obesity, abdominal obesity, body mass index, waist to hip ratio, diabetic retinopathy, cohort

## Abstract

**Background:**

The relationship between obesity and diabetic retinopathy (DR) remains controversial. The aim of this study was to assess the association of generalized obesity [assessed by body mass index (BMI)] and abdominal obesity [assessed by waist to hip ratio (WHR)] with incident DR, and vision-threatening DR (VTDR), and DR progression among Chinese adults with type 2 diabetic mellitus (T2DM).

**Method:**

This prospective cohort study was conducted at the Zhongshan Ophthalmic Center, from November 2017 to December 2020. DR was assessed based on the 7-filed fundus photographs using the modified Airlie House Classification. Multivariable logistic regression models were used to evaluate the associations of BMI and WHR with the development and progression of DR after adjusting for age, sex, traditional risk factors, and mutually for BMI and WHR.

**Results:**

Among the 1,370 eligible participants, 1,195 (87.2%) had no sign of any DR and 175 (12.8%) had DR at baseline examination. During the 2 years follow-up visit, 342 (28.6%) participants had incident DR, 11 (0.8%) participants developed VTDR, 15 (8.6%) demonstrated DR progression. After adjusting for confounders, the BMI was negatively associated with incident DR [relative risk (RR) =0.31; 95% confidence interval (CI), 0.26–0.38; *P* < 0.001] and incident VTDR (RR = 0.22; 95%CI, 0.11–0.43; *P* < 0.001), while WHR was positively associated with incident DR (RR = 1.47; 95% CI, 1.27–1.71; *P* < 0.001). BMI and WHR level were not significantly associated with 2-year DR progression in multivariate models (all *P* > 0.05).

**Conclusions:**

This study provides longitudinal evidence that generalized obesity confer a protective effect on DR, while abdominal obesity increased the risk of DR onset in Chinese patients, indicating that abdominal obesity is a more clinically relevant risk marker of DR than generalized obesity.

## Introduction

As a common microvascular complication of diabetes mellitus (DM), diabetic retinopathy (DR) is the leading cause of blindness among working-age adults worldwide, which may lead to poor quality of life and increased socioeconomic burden (1, 2). The numbers of DR are expected to increase greatly over the next decade with the increased prevalence of DM and improved lifestyle, and it is estimated that it will reach to 191 million for any DR and 56.6 million for vision-threatening diabetic retinopathy (VTDR) by 2030 without prompt intervention and treatment (3). Therefore, strategies to reduce the burden of DR is pressing and studies to identify modifiable risk factors of DR are essential to guide clinical practice to prevent DR occurrence and progression (4).

Body mass index (BMI), which represent generalized obesity, is the most commonly-used anthropometric measurement to assess the relationship between overweight/obesity and DR, but their associations remain inconsistent, with some studies indicated a positive association (5–7), while others indicated negative association (8–10), or no association (11, 12). It was also reported that a U-shaped relationship between BMI and DR among type 2 diabetes patients (13). Recent data suggested that distribution of adipose tissue rather than the amount might play a key role in association of obesity with diabetic microvascular complications (14, 15). Anthropometric indices that use waist to hip ratio (WHR) to assess abdominal obesity, may be a better marker in predicting the risk of DR. However, previous studies on associations of WHR and DR were also controversy (8–10).

Currently, studies on obese and DR were mainly in cross-sectional design, longitudinal cohort studies are limited and inconclusive. To our knowledge, there were no longitudinal cohort studies on association of WHR with DR. To address these gaps, the purpose of this study was to evaluate the relationship between different kind of obesity with incident DR, vision-threatening diabetic retinopathy (VTDR) and DR progression in a prospective cohort of Chinese individuals with T2DM.

## Methods

### Study Participants

This is a community-based prospective cohort study conducted at Zhongshan Ophthalmic Center, Sun Yat-sen University, China. The study adhered to the Declaration of Helsinki and was approved by the Institute Ethics Committee of ZOC (2017KYPJ094). Each participant provided written informed consent. The detailed methodology has been descripted previously (16). In brief, 2011 participants aged 30–80 years who were diagnosed with T2DM without ocular treatment were recruited from a community in the Yuexiu district, Guangzhou from November 2017 to October 2019. All participants underwent detailed examinations at baseline examination, and were followed annually using the same study protocol. This study analyzes the data collected at 2-year follow-up examinations.

The inclusion criteria for our study participants were as follows: (1) type 2 DM and aged 30–80 years, (2) no history of ocular treatment (ocular treatment naïve), (3) visual acuity of 0.1 or more and able to complete an eye examination, and (4) spherical degree of >−6 diopters (D), astigmatism of <1.5 D, and axial length (AL) of <26 mm. Participants were excluded in the presence of any of the following conditions: (1) history of serious systemic diseases other than diabetes, such as uncontrolled hypertension, serious cardiovascular and cerebrovascular diseases, malignant tumor, or nephritis; (2) history of systemic surgery, thrombolysis therapy, or renal dialysis; (3) glaucoma, vitreous-macular diseases (vitreous hemorrhage and retinal detachment), or amblyopia; (4) history of retina laser or intraocular injection, glaucoma surgery, cataract surgery, or corneal refractive surgery; and (5) poor quality of fundus images resulting from abnormal refractive media (such as moderate to severe cataract, corneal ulcer, or severe pterygium), poor fixation or other causes. Participants who had baseline VTDR, lost to follow-up, had intraocular surgery at either eye during 2-year follow-up period, and those with missing data were excluded from the current study.

### Study Examinations

All participants underwent a standardized questionnaire, physical and ocular examinations at baseline and each follow-up examination using the same study protocol. A standardized questionnaire was used by a trained interviewer to collect information of age, duration of diabetes, systemic chronic diseases and ocular disease, medication usage and history of systemic and ocular surgery. A brief questionnaire of self-reported change in chronic disease status (disease and medicine) was also conducted at each follow-up. Systolic blood pressure (SBP) and diastolic blood pressure (DBP) were measure using a digital BP monitor (Hem-907, Omron, Kyoto, Japan) by the same trained personnel. Venous blood samples were collected to assess total cholesterol, high-density lipoprotein cholesterol (HDL), low-density lipoprotein cholesterol (LDL), triglycerides (TG), serum creatinine (Scr) and hemoglobin A1c (HbA1c) by standardized methods. All participants underwent detailed ocular examination followed standard protocol, including uncorrected visual acuity (UCVA), best corrected visual acuity (BCVA), non-cycloplegic refractions (KR8800; Topcon, Tokyo, Japan), ocular biometry (Lenstar, LS900, Haag-Streit, Koeniz, Switzerland) and non-contact tonometer (CT-1, Topcon, Tokyo, Japan).

### Assessment of BMI and WHR

Height and weight were measured in standing position using an automatic scale (HNH-318; OMRON) without shoes and heavy clothes on. BMI was calculated as weight in kilograms (kg) divided by height in meters squared (m^2^), and was categorized into three group: underweight/normal (<23 kg/m^2^), overweight (23–27.5 kg/m^2^), and obese (≥27.5 kg/m^2^), according to WHO-recommended Asian cut points for obesity (17). A non-stretchable medical tape was used to measure waist and hip circumference (in centimeters). Waist circumference was measured at the smallest horizontal girth between the costal margins and the iliac crests at the end of tidal expiration, while hip measurement at the maximal protuberance of the buttocks. WHR was calculated by dividing the waist circumferences by the hip circumferences.

### Assessment of DR and Definition of End Points

Standard ETDRS 7-field fundus photography were taken for each eye using a digital fundus camera (Canon CR-2, Canon, Tokyo, Japan) after pupil dilation (0.5% tropicamide plus 0.5% phenylephrine). The presence and severity of DR were graded by two well-trained graders in according to the Modified Airlie House Classification system (18). Any DR was defined as the presence of non-proliferative DR (NPDR), proliferative DR(PDR), diabetic macular oedema (DME), or any combination at any eye. VTDR was defined as the presence of PDR and/or DME. The DR level for each participant was then derived by concatenating the levels for the two eyes, giving greater weightage to the eye with the more severe grade. Incident DR was defined as participants without baseline DR who developed DR at any eye during the 2-year follow-up period. Incident VTDR was defined as development of VTDR at 2-year follow-up examination with no sign of VTDR at baseline. Progression of DR was defined as any two level or greater worsening of DR at 2 years (19, 20).

### Statistical Analyses

Statistical analyses were performed using Stata (Ver.16.0; Stata Corporation, College Station, Texas, USA). BMI was analyzed continuously and further categorized into three groups based on the WHO-recommended Asian BMI classification system. And WHR was evaluated continuously and categorically in three tertiles. Continuous variables were presented as mean ± standard deviation (SD) and categorical variables as proportion. Student's *t*-test for continuous variables and χ2 test for categorical variables were used to compare the characteristics of participants with and without incident DR, incident VTDR or DR progression. Logistic regression models were used to assess the association of obesity index (BMI and WHR), both as continuous and categorical variables, with incident DR, VTDR and DR progression with two models. Model 1 was adjusted for age and gender, and model 2 further adjusted for HbA1c, duration of diabetes, use of insulin, SBP, DBP, total cholesterol, and triglycerides respectively. *P*-values of <0.05 were considered statistically significant.

## Results

**Figure 1:**
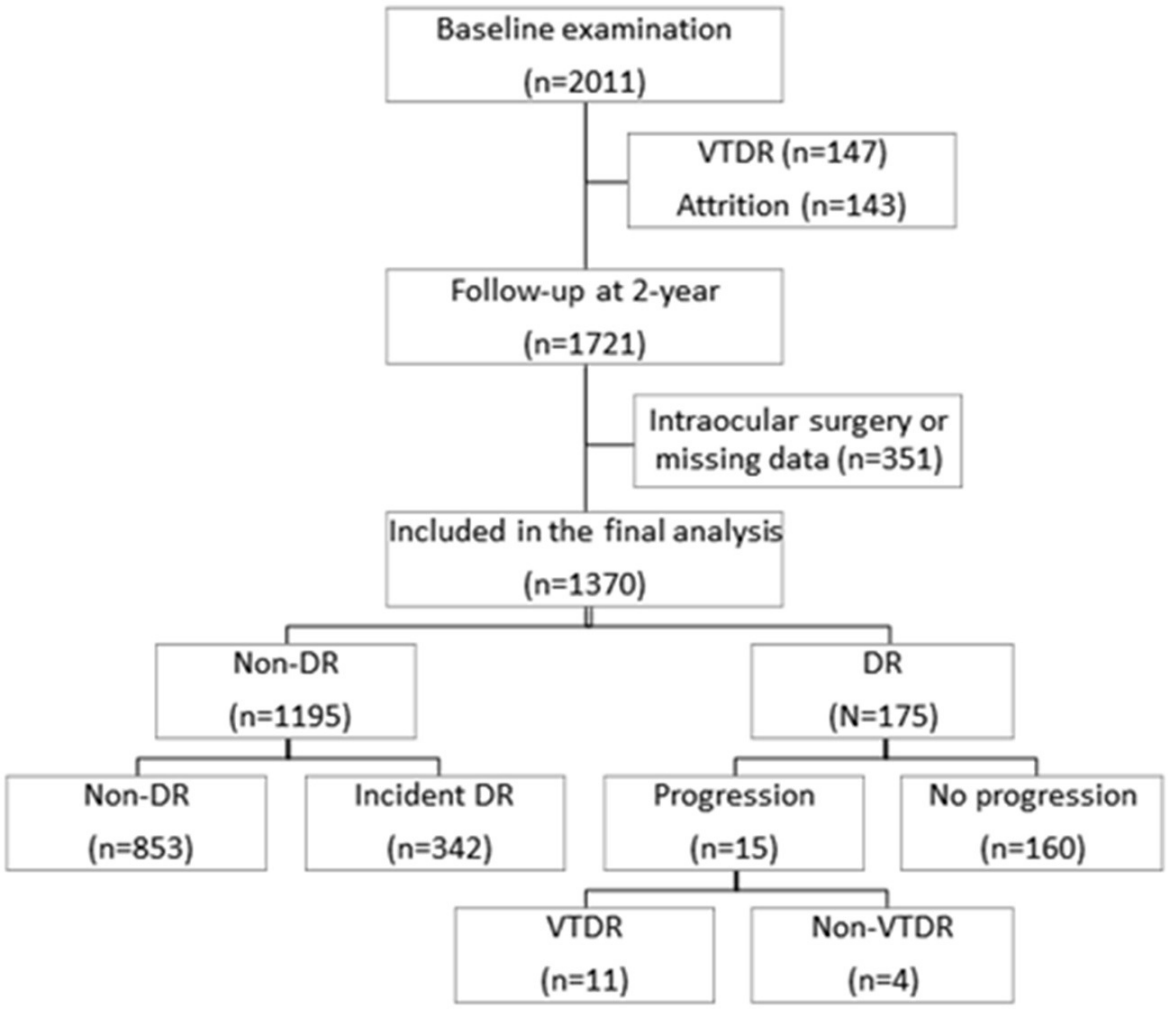
Flowchart of the participants selection.

Figure 1 shows the flowchart of the diabetic participants during the 2 years follow-up. Among the 2011 participants at baseline, a total of 1,721 (85.6%) attended the 2-year follow-up examination. Two hundred and ninety participants were excluded for 147 (7.3%) with baseline VTDR and 143 (7.3%) individuals lost at follow-up. Of the 1,721 participants who attended 2-year follow-up examination, we further excluded 351 (31.9%) participants who had undergone intraocular surgery or missing data, leaving 1,370 (68.1%) participants eligible for the final analysis. No significant differences were found in baseline demographics and DR severity between the 1,370 included participants with the 351 excluded participants (all *P* > 0.05).

Among the 1,370 eligible participants, 1,195 (87.2%) had no sign of any DR and 175 (12.8%) had DR at baseline examination. During the 2 years follow-up visit, 342 (28.6%) of the 1,195 participants had incident DR, 11 (0.8%) of the 1,370 diabetic patients developed VTDR, and 15 (8.6%) of the 175 participants with baseline DR demonstrated DR progression. The baseline demographic and clinical characteristics of the study participants are showed in Table 1. Participants with incident DR were more likely to be younger, not use insulin, have higher HbA1c level, have a lower BMI level, have larger WHR, and have lower HDL cholesterol level (all *P* < 0.05) compared with those without incident DR. Participants with incident VTDR tended to have longer duration of diabetes, higher HbA1c level and lower BMI level (all *P* < 0.001). Compared to participants without DR progression, those with 2-year DR progression were more likely to be female, have higher levels of HbA1c and lower BMI level (all *P* < 0.05).

**Table 1:** Baseline demographic and clinical features of the included participants.

**Characteristics**	**Incident DR**			**Incident VTDR**			**DR progression**		
	**No**	**With**	***P***	**No**	**With**	***P***	**No**	**With**	***P***
*N*	853 (71.4%)	342 (28.6%)	–	1,363 (99.2%)	11 (0.8%)	–	160 (91.4%)	15 (8.6%)	–
Female, %	493 (57.8%)	199 (58.2%)	0.901	770 (56.5%)	9 (81.8%)	0.091	75 (46.9%)	12 (80.0%)	**0.014**
Use of insulin, %	138 (40.4%)	77 (9.0%)	**0.010**	274 (20.1%)	4 (36.4%)	0.181	56 (35.0%)	5 (33.3%)	0.897
Age, year	65.0 ± 8.04	62.9 ± 8.64	**<0.001**	64.3 ± 8.15	64.6 ± 7.67	0.917	63.8 ± 8.01	61.9 ± 8.42	0.397
Duration of diabetes, year	8.33 ± 6.52	8.86 ± 6.85	0.208	8.94 ± 6.85	16.1 ± 5.47	**<0.001**	12.4 ± 7.70	14.1 ± 5.74	0.399
HbA1c, %	6.61 ± 1.04	7.06 ± 1.40	**<0.001**	6.83 ± 1.25	8.85 ± 1.72	**<0.001**	7.49 ± 1.54	8.47 ± 1.75	**0.020**
Body mass index, kg/m^2^	25.9 ± 2.49	23.7 ± 2.21	**<0.001**	25.1 ± 2.69	21.6 ± 2.88	**<0.001**	24.2 ± 3.01	22.1 ± 2.71	**0.010**
Waist to hip ratio	0.88 ± 0.08	0.90 ± 0.07	**<0.001**	0.89 ± 0.08	0.90 ± 0.05	0.738	0.91 ± 0.06	0.90 ± 0.05	0.582
SBP, mmHg	131.5 ± 18.2	132.2 ± 18.1	0.532	132.3 ± 18.3	142.1 ± 19.2	0.079	136.2 ± 19.1	141.3 ± 21.9	0.335
DBP, mmHg	70.2 ± 10.0	71.1 ± 10.1	0.165	70.4 ± 10.0	68.0 ± 10.0	0.431	69.2 ± 9.7	71.4 ± 11.6	0.418
Total cholesterol, mmol/L	4.80 ± 1.09	4.70 ± 0.95	0.133	4.77 ± 1.07	4.98 ± 1.04	0.516	4.81 ± 1.19	5.15 ± 0.94	0.296
Triglycerides, mmol/L	2.38 ± 1.68	2.34 ± 1.72	0.722	2.39 ± 1.71	1.85 ± 0.82	0.298	2.47 ± 1.87	2.26 ± 1.02	0.681
HDL-c, mmol/L	1.30 ± 0.40	1.25 ± 0.36	**0.035**	1.29 ± 0.39	1.50 ± 0.36	0.067	1.29 ± 0.37	1.38 ± 0.37	0.373

*SBP, systolic blood pressure; DBP, diastolic blood pressure; DR, diabetic retinopathy; VTDR, vision threatening diabetic retinopathy; HDL-c, high-density lipoprotein cholesterol. Bold indicates statistical significance*.

*All data presented as mean ± standard deviation unless otherwise indicated*.

### Associations of BMI and WHR With Incident DR

In multivariable logistic regression model 1, we observed that overweight [relative risk (RR), 0.28; 95% CI, 0.20–0.40; *P* < 0.001] and obesity (RR, 0.15; 95% CI, 0.11–0.20; *P* < 0.001) were associated with lower risk of incident DR. This inverse association persisted when BMI was analyzed as a continuous variable (RR, 0.31; 95% CI, 0.26–0.38, *P* < 0.001). After additionally adjusting for other confounders in model 2, the inverse relationship of the BMI-DR association remained unchanged both in continuous and categorical analysis (all *P* < 0.05).

Participants in the highest tertiles of WHR measurements were more likely to have incident DR compared with those participants in the lowest WHR tertiles in multivariable logistic regression model 1 (RR, 1.61; 95% CI, 1.18–2.21; *P* = 0.003) and model 2 (RR, 1.59; 95% CI, 1.15–2.21; *P* = 0.005). This positive association persisted when WHR was analyzed as a continuous variable in model 1 (RR, 1.47; 95% CI, 1.27–1.71; *P* < 0.001) and model 2 (RR, 1.49; 95% CI, 1.27–1.74; *P* < 0.001) (Table 2).

**Table 2:** Associations between BMI and WHR with the incidence of DR.

	Model 1^*^		**Model 2^†^**	
	**RR (95%CI)**	***P***	**RR (95%CI)**	***P***
**BMI categories**				
Normal/underweight	1.0 (Reference)		1.0 (Reference)	
Overweight	0.28 (0.20–0.40)	**<0.001**	0.28 (0.20–0.40)	**<0.001**
Obese	0.15 (0.11–0.20)	**<0.001**	0.14 (0.10–0.20)	**<0.001**
BMI per 1-SD increase	0.31 (0.26–0.38)	**<0.001**	0.31 (0.25–0.37)	**<0.001**
**WHR categories**				
Tertile 1	1.0 (Reference)		1.0 (Reference)	
Tertile 2	1.05 (0.77–1.45)	0.754	1.02 (0.73–1.42)	0.905
Tertile 3	1.61 (1.18–2.21)	**0.003**	1.59 (1.15–2.21)	**0.005**
WHR per 1-SD increase	1.47 (1.27–1.71)	**<0.001**	1.49 (1.27–1.74)	**<0.001**

*BMI, body mass index; WHR, waist to hip ratio; SD, standard deviation; DR, diabetic retinopathy; RR, relative risk; CI, confidence interval*.

* *Adjusted for age and sex*.

† *Additionally adjusted for HbA1c, duration of diabetes, use of insulin, SBP, DBP, total cholesterol, and triglycerides. Bold indicates statistical significance*.

### Associations of BMI and WHR With Incident VTDR

Higher BMI was significantly associated with decreased risk of incident VTDR both in multivariable model 1(RR, 0.22; 95% CI, 0.11–0.43; *P* < 0.001) and model 2(RR, 0.37; 95% CI, 0.17–0.78; *P* = 0.009). When BMI were analyzed as categorical variables, individuals with overweight (RR, 0.12; 95% CI, 0.01–0.96; *P* = 0.046) or obesity (RR, 0.12; 95% CI, 0.03–0.57; *P* = 0.008) were less likely to have incident VTDR compared with normal/underweight participants in multivariable model 1. This association between overweight (RR, 0.18; 95% CI, 0.02–1.57; *P* = 0.120) or obesity (RR, 0.26; 95% CI, 0.05–1.39; *P* = 0.116) with risk for incident VTDR was not statistically significant in multivariable model 2. WHR, both as continuous and categorical variable, was not associated with incident VTDR in both multivariable models (all *P* > 0.05) (Table 3).

**Table 3:** Associations between BMI and WHR with the incidence of VTDR.

	Model 1^*^		**Model 2^†^**	
	**RR (95%CI)**	***P***	**RR (95%CI)**	***P***
**BMI categories**				
Normal/underweight	1.0 (Reference)		1.0 (Reference)	
Overweight	0.12 (0.01–0.96)	**0.046**	0.18 (0.02–1.57)	0.120
Obese	0.12 (0.03–0.57)	**0.008**	0.26 (0.05–1.39)	0.116
BMI per 1-SD increase	0.22 (0.11–0.43)	**<0.001**	0.37 (0.17–0.78)	**0.009**
**WHR categories**				
Tertile 1	1.0 (Reference)		1.0 (Reference)	
Tertile 2	1.57 (0.35–7.14)	0.556	2.82 (0.48–16.43)	0.250
Tertile 3	1.85 (0.40–8.58)	0.434	3.36 (0.55–20.36)	0.187
WHR per 1-SD increase	1.26 (0.63–2.52)	0.519	1.78 (0.69–4.60)	0.230

*BMI, body mass index; WHR, waist to hip ratio; SD, standard deviation; VTDR, vision threatening diabetic retinopathy; RR, relative risk; CI, confidence interval*.

* *Adjusted for age and sex*.

† *Additionally adjusted for HbA1c, duration of diabetes, use of insulin, SBP, DBP, total cholesterol, and triglycerides. Bold indicates statistical significance*.

### Associations of BMI and WHR With DR Progression

Higher BMI was associated with a lower likelihood of having DR progression (RR, 0.52; 95% CI, 0.29–0.93, *P* = 0.028) after adjusting for age and gender (model 1), but this inverse association was no longer statistically significant after further adjusting for confounders in model 2 (RR, 0.63; 95% CI, 0.32–1.21, *P* = 0.163). WHR was not associated with DR progression in in both multivariable models (all *P* > 0.05) (Table 4).

**Table 4:** Associations between BMI and WHR with the presence of DR progression.

	Model 1^*^		**Model 2^†^**	
	**RR (95%CI)**	***P***	**RR (95%CI)**	***P***
**BMI categories**				
Normal/underweight	1.0 (Reference)		1.0 (Reference)	
Overweight	0.49 (0.12–2.00)	0.318	0.63 (0.13–3.02)	0.567
Obese	0.37 (0.09–1.48)	0.159	0.45 (0.10–2.14)	0.317
**WHR categories**				
Tertile 1	1.0 (Reference)		1.0 (Reference)	
Tertile 2	1.20 (0.30–4.81)	0.795	2.67 (0.43–16.68)	0.293
Tertile 3	1.07 (0.25–4.64)	0.927	2.79 (0.42–18.62)	0.290
BMI per 1-SD increase	0.52 (0.29–0.93)	**0.028**	0.63 (0.32–1.21)	0.163
WHR per 1-SD increase	1.06 (0.46–2.42)	0.898	1.82 (0.68–4.84)	0.232

*BMI, body mass index; WHR, waist to hip ratio; SD, standard deviation; DR, diabetic retinopathy; RR, relative risk; CI, confidence interval*.

* *Adjusted for age and sex*.

† *Additionally adjusted for HbA1c, duration of diabetes, use of insulin, SBP, DBP, total cholesterol, and triglycerides. Bold indicates statistical significance*.

## Discussion

The current study investigated the association between generalized obesity (assessed using BMI) and abdominal obesity (WHR) with the incidence and progression of DR in a China population with T2DM. We found that higher BMI was associated with decreased risk of incident DR and VTDR, while WHR was associated with increased risk of incident DR during a 2-year follow-up.

Although numbers of cross-sectional and longitudinal epidemiological and clinical studies have investigated the association between BMI and DR, the results were still equivocal, which could be owing to the different in study design, region, ethnicity (8). For instance, most obervations of protective effect against the incidence of DR were performed in Asians (9, 10, 21). The current study also indicated that higher BMI play a protective role in incident DR and VTDR in Chinese T2DM. Several possible explanations underlying protect effect of BMI on DR. Firstly, higher BMI is associated with higher fasting C-peptide which may decrease the risk of DR (22, 23). Secondly, this protective effect may due to the “obesity paradox”, which means that participants with overweight/obese may had more intensive treatment for other comorbidities and simultaneously result in better health outcome including decreasing the risk of DR or other complications (24). Finally, patients with uncontrolled diabetes with concomitant comorbidities may have lower BMI because of unintentional weight loss (10).

Existed evidence for WHR-DR association is limited. There were inconsistencies in cross-sectional studies for WHR-DR association. For instance, the Singapore Diabetes Management Project study reported higher WHR was associated with the presence of any DR, but association of WHR-DR severity was observed in women only (10). The SN-DREAMS-I Study also demonstrated a significant association between increased WHR and risk of having DR in women among Indian adults (25). In contrast, some studies conducted in China have reported null WHR-DR associations (11, 14). It should be noted that all aforementioned studies were cross-sectional study, and the causal relationship cannot be determined. To the best of our knowledge, this is the first longitudinal cohort study to investigate the relationship between abdominal obesity and DR. Our results showed that higher WHR increased the risk of 2-year incident DR, highlighting that WHR may be a potentially more clinically relevant marker in the pathogenesis of DR than BMI.

The mechanisms underlying the detrimental WHR-DR association remain unclear. One possible reason is that the excess fat deposition in the abdominal region caused a higher release of free fatty acids in circulation which play a central role in insulin resistance, hyperlipidemia, inflammation, hypertension, all of which are known to be established factors pathogenesis of DR (26). Moreover, release of excess free fatty acids and adipokines by excess abdominal fat cause oxidative stresses, choronic systematic inflammation, abnormal endothelial function, favoring diabetic retinal microangiopathy development (27). Additionally, abdominal obesity had an inhibitory effect on the spontaneous pulsatile secretion of growth hormone (GH), which has been proved to be significantly related to adverse metabolic complication (28, 29).

The relationship between obesity and DR progression remains elusive. The Singapore Indian Eye study indicated that BMI were associated with lower incident DR but associated with higher risk of DR progression among Indian diabetic adults over 6-year periods (30), while the Sankara Nethralaya-Diabetic Retinopathy Epidemiology and Molecular Genetics Study (SN-DREAMS II) indicated an inverse association between BMI and DR progression in Indian adults over 4-year periods (25). The Wisconsin Epidemiologic Study of DR reported a non-significant association of BMI-DR progression in their 10-year follow-up visit, but a positive association of BMI-DR progression was detect in their 25-year follow-up visit in persons with type 1 DM. Our study did not detected significantly associations between obesity and DR-progression in multivariable models. The relatively small cases of baseline DR and DR progression, as well as the shorter follow-up time may partly explain the non-associations in the current study. Further longitudinal studies with larger sample size are warranted.

The strengths of current study lie in that longitudinal lcohort study based in a single Chinese community, adjustment of several potential confounders and the larger study sample size with detailed systemic and ocular examinations followed the same study protocol. The 7-field fundus photograph enable the maximum possibility to detect signs of DR in the peripheral retina. However, there are some limitations in our study. Firstly, all participants in the current study were recruited from communities, thus our findings cannot be generalized to other areas or races. Secondly, the relatively shorter follow-up periods may limit the analysis of relationship between obese and incident DR. Thirdly, only Chinese patients were included, however, ethnic differences of DR occurrence and progressions were reported. Thus, the generalization of the findings to other ethnicity should be taken cautions.

## Conclusions

In summary, we found that higher BMI was associated with a lower incidence of DR and VTDR over a 2-year period, but a higher WHR is associated with increased likelihood of the DR in this large cohort study, highlighting the fact that abdominal obesity is a more clinically relevant risk marker of DR than generalized obesity for individuals with type 2 DM. Further studies with bigger sample size and longer follow-up period are warranted to confirm the predictive effect of anthropometric measurement on onset and progression of DR.

## Data Availability Statement

The raw data supporting the conclusions of this article will be made available by the corresponding authors on reasonable request.

## Ethics Statement

The studies involving human participants were reviewed and approved by Zhongshan Ophthalmic Center (2017KYPJ094). The patients/participants provided their written informed consent to participate in this study.

## Author Contributions

LW, XL, XH, and WH: design and conduct of the study. XH, XL, YL, MY, XGo, RT, and MX: collection, management, analysis, and interpretation of the data. XH, HW, XL, XGu, LW, and WH: preparation of the manuscript. All authors: review and final approval of the manuscript.

## Funding

This research was supported by the National Natural Science Foundation of China (82171084), the Fundamental Research Funds of the State Key Laboratory of Ophthalmology (303060202400362). The funder had no role in the design and conduct of the study, collection, management, analysis, and interpretation of the data, preparation, review, or approval of the manuscript, and the decision to submit the manuscript for publication.

## Conflict of Interest

The authors declare that the research was conducted in the absence of any commercial or financial relationships that could be construed as a potential conflict of interest. The handling editor ZL declared a shared affiliation with the authors XGu, XGo, XL, WH, MY, and LW at the time of review.

## Publisher's Note

All claims expressed in this article are solely those of the authors and do not necessarily represent those of their affiliated organizations, or those of the publisher, the editors and the reviewers. Any product that may be evaluated in this article, or claim that may be made by its manufacturer, is not guaranteed or endorsed by the publisher.
